# Scalable Production in Human Cells and Biochemical Characterization of Full-Length Normal and Mutant Huntingtin

**DOI:** 10.1371/journal.pone.0121055

**Published:** 2015-03-23

**Authors:** Bin Huang, Tanja Lucas, Claudia Kueppers, Xiaomin Dong, Maike Krause, Alexander Bepperling, Johannes Buchner, Hans Voshol, Andreas Weiss, Bertran Gerrits, Stefan Kochanek

**Affiliations:** 1 Department of Gene Therapy, Ulm University, Ulm, Germany; 2 Center for Integrated Protein Science Munich, Department of Biotechnology, Technische Universität München, Garching, Germany; 3 Novartis Institute for Biomedical Research, Basel, Switzerland; Institut Curie, FRANCE

## Abstract

Huntingtin (Htt) is a 350 kD intracellular protein, ubiquitously expressed and mainly localized in the cytoplasm. Huntington’s disease (HD) is caused by a CAG triplet amplification in exon 1 of the corresponding gene resulting in a polyglutamine (polyQ) expansion at the N-terminus of Htt. Production of full-length Htt has been difficult in the past and so far a scalable system or process has not been established for recombinant production of Htt in human cells. The ability to produce Htt in milligram quantities would be a prerequisite for many biochemical and biophysical studies aiming in a better understanding of Htt function under physiological conditions and in case of mutation and disease. For scalable production of full-length normal (17Q) and mutant (46Q and 128Q) Htt we have established two different systems, the first based on doxycycline-inducible Htt expression in stable cell lines, the second on “gutless” adenovirus mediated gene transfer. Purified material has then been used for biochemical characterization of full-length Htt. Posttranslational modifications (PTMs) were determined and several new phosphorylation sites were identified. Nearly all PTMs in full-length Htt localized to areas outside of predicted alpha-solenoid protein regions. In all detected N-terminal peptides methionine as the first amino acid was missing and the second, alanine, was found to be acetylated. Differences in secondary structure between normal and mutant Htt, a helix-rich protein, were not observed in our study. Purified Htt tends to form dimers and higher order oligomers, thus resembling the situation observed with N-terminal fragments, although the mechanism of oligomer formation may be different.

## Introduction

Huntington’s disease (HD) is an inherited neurodegenerative disorder with preferential neuronal cell loss in the striatum and the cortex that is characterized by abnormal cytoplasmic and nuclear aggregates at the microscopic level [1–4]. The clinical features of HD are well known and include progressive motoric dysfunction, cognitive decline and psychiatric disturbances [5]. HD is caused by an increased number (≥36) of consecutive CAG trinucleotide repeats in the exon 1 region of the HD gene that upon translation result in a polyglutamine (polyQ) expansion at the N-terminus of the protein Huntingtin (Htt) [6]. Full penetrance in HD is observed with alleles of ≥40 repeats and reduced penetrance with alleles of between 36 and 39 repeats [7–9]. Most published data suggest mainly a toxic gain-of-function of mutant Htt and Htt fragments [10–13]. This then causes the disease with additional evidence also for a contribution by loss-of-function mechanisms [14, 15]. Many mechanisms have been proposed to explain the observed morphological and molecular abnormalities observed in HD including generation of toxic Htt fragment species, excitotoxicity, energy deficiency and others [16, 17]. However, a detailed understanding of the pathogenesis of HD at the molecular level is still lacking.

With a molecular weight (MW) of about 350 kD Htt is a very large intracellular protein that is mainly localized in the cytoplasm. It is likely involved in many different global cellular functions such as gene expression, vesicle trafficking, endocytosis, intracellular signaling and metabolism [18–22].

Sequence comparisons via homology searches with proteins of known function have not resulted in specific information useful for the prediction of Htt domain functions. An important finding, however, has been the observation that Htt contains a large number of HEAT repeat motifs, pairs of antiparallel α-helices with a length of about 40 amino acids, that have been observed in several proteins in addition to Htt including importin-β, karyopherin-β2, PP2A and Cand1 [23, 24]. These HEAT repeat rich proteins are predicted to have a high degree of conformational flexibility and are thus predestined to function, for example, as scaffolding proteins. After HEAT repeats had been recognized as a specific structural entity [23], Takano and Gusella suggested that Htt contains about 28–36 HEAT repeats that may favor the formation of dynamic complexes with protein partners analogous to other HEAT-rich proteins [25].

A bottleneck for biochemical and biophysical characterisation of full-length normal and mutant Htt has been the lack of a scalable production system. Li et al [26] and Seong et al [27] described production of Htt in insect cells using the Baculovirus production system observing μg levels of soluble protein.

We have generated two systems for recombinant production of full-length normal and mutant Htt in human cells. The first system is based on stable cell lines expressing normal or mutant Htt under doxycycline-inducible expression control. The second system utilizes high-capacity adenovirus (HC-Ad) vector technology (also called helper-dependent or “gutless” Ad vectors) for gene transfer in human cells. A two-step purification process was established based on affinity chromatography followed by size exclusion chromatography (SEC) resulting in highly purified Htt. Analysis by mass spectrometry (MS) of normal and mutant Htt lead to the identification of both known and novel phosphorylation sites. Further biochemical characterization of this human cell derived full-length Htt confirmed that it is a helix-rich and non-globular protein and that it is prone to form dimers and oligomers.

## Materials and Methods

### Antibodies

Antibodies used in this study, along with the epitopes recognized by the respective antibody and vendor, were monoclonal antibody 2B4 (Htt 115–129, Merck Millipore), monoclonal antibody MAB2166 (Htt 443–457, Merck Millipore) and Anti-FLAG M2 antibody (Sigma).

### Generation of the Htt expressing cell lines B1.21 and C2.6

DNA constructs were generated containing a doxycycline (Dox)-inducible promoter, a cDNA coding for either human full-length normal Htt with 17Qs (Htt17) or coding for full-length mutant Htt with 46Qs (Htt46), both c-terminally fused with a FLAG-His affinity tag, and followed by the SV40 late polyadenylation signal (SV40 pA). The Htt-SV40pA constructs were cloned into the vector pTRE-Tight-BI-AcGFP1 (Clontech) that allowed co-expression of Htt with GFP upon induction with Dox. The resulting plasmid constructs pTL6 (coding for Htt17) and pTL8 (coding for Htt46) were verified by restriction analysis and by partial sequencing. HEK293 Tet-ON cells (Clontech) were co-transfected with linearized pTL6 or pTL8 and a linearized plasmid expressing a hygromycin (Hyg) resistance gene. Positive cell clones were selected by addition of Hyg to the culture medium. Monoclonal cell lines B1.21 (expressing Htt17) and C2.6 (expressing Htt46) were obtained via limited dilution of positive cell clones.

### Generation of HC-Ad vectors HC-Ad-Htt17Q-FH and HC-Ad-Htt128Q-FH

**Fig 1:**
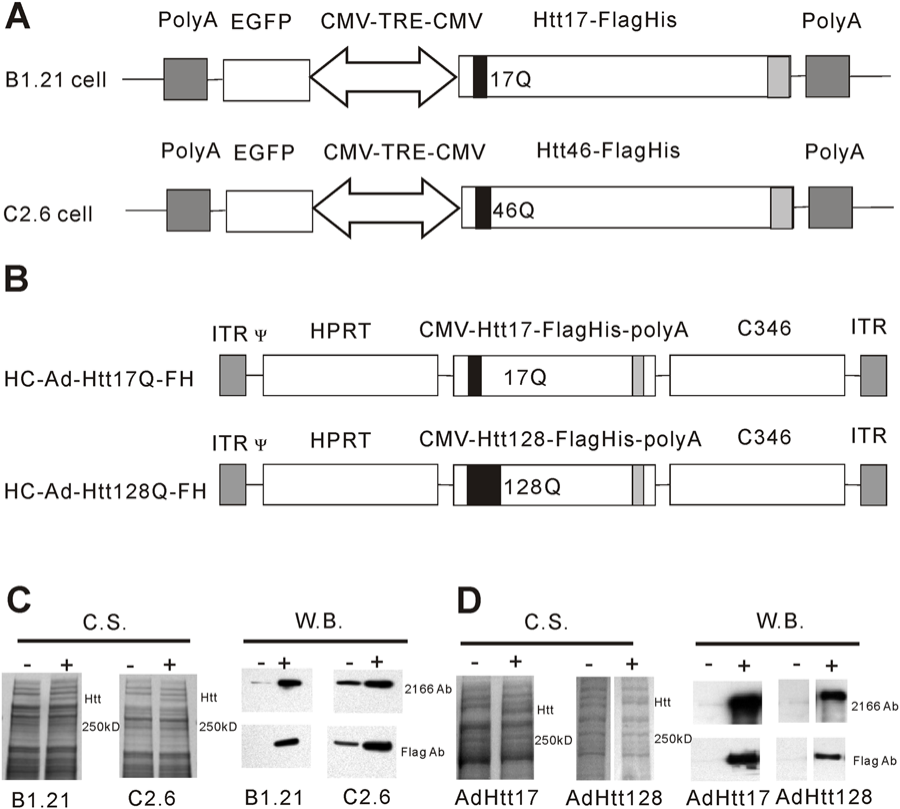
Production of full-length normal and mutant Htt in stable cell lines and following adenovirus-mediated gene transfer. (A) Constructs for expression of Htt17 in B1.21 cells and for expression of Htt46 in C2.6 cells. Htt17 and Htt46 are c-terminally fused to a FLAG-His affinity tag and are expressed under the control of a Dox-inducible and bidirectional minimal CMV promoter tetracycline responsive element (CMV-TRE-CMV). (B) Adenovirus vector constructs for expression of Htt17 (HC-Ad-Htt17Q-FH) and of Htt128 (HC-Ad-Htt128Q-FH). Htt17 and Htt128 are c-terminally fused to a FLAG-His tag and are expressed under control of the CMV promoter. HC-Ad-Htt16Q-FH and HC-Ad-Htt128Q-FH vectors also contain stuffer DNAs (hprt and c346) from human genomic DNA, the inverted terminal repeats (ITR) of hAd5 and the packaging signal of hAd5 (Ψ). (C) The expression of Htt17 and Htt46 in B1.21 and C2.6 cells was analyzed 48 hours after induction with Dox by SDS-PAGE and Coomassie Blue staining (C,S.) and was further confirmed by Western Blot (W.B.) analysis. Compared to un-induced cells (-), an additional band of about 350 kD size was clearly visible in cells 48 hours after induction (+). 2166 Ab: antibody recognizing Htt; Flag Ab: antibody recognizing the FLAG tag. (D) Expression of Htt17 and Htt128 following transduction of 293 cell-based 116 cells with HC-Ad-Htt17Q-FH and HC-Ad-Htt128Q-FH vector was analyzed 48 hours after gene transfer by SDS-PAGE and Coomassie Blue staining and was further confirmed by Western Blot analysis. Compared with un-transduced cells (-), additional bands of about 350 kD size and reacting with anti-Htt and anti-FLAG antibodies are visible following HC-Ad vector mediated transduction (+).

An expression cassette was generated consisting of the cytomegalovirus early promoter/enhancer (CMV promoter), an intron, the cDNA coding for human full-length mutant Htt with either 17Qs or 128Qs c-terminally fused with a FLAG-His (FH) affinity tag, and the SV40pA signal. The expression cassette was inserted into the SwaI site of the HC-Ad vector shuttle plasmid pSTK119 resulting in plasmid pSTK119-Htt128Q-FH. The HC-Ad vectors HC-Ad-Htt17Q-FH and HC-Ad-Htt128Q-FH were produced as described previously [28]. Briefly, pSTK119-Htt17Q-FH and pSTK119-Htt128Q-FH were cleaved with PmeI followed by transfection into 116 cells [29]. The cells were subsequently infected with the helper virus AdLC8cluc [30]. Production and purification followed published protocols [31]. Infectious and particle titers were determined by the slot blot method [32]. The integrity of the vector genomes was confirmed by restriction analysis. The maps of HC-Ad-Htt17Q-FH and HC-Ad-Htt128Q-FH are shown in Fig. 1B.

### Coomassie Blue staining and Western Blot analysis

Coomassie Blue staining and Western blot analysis were performed according to standard protocols [33]. In brief, cell lysates or purified proteins were denatured in sample buffer (50 mM Tris HCl, 0.1 M DTT, 2% SDS, 0.1% bromophenol blue and 10% glycerol, pH 6.8) at 98°C for 5 min, followed by sodium dodecyl sulfate polyacrylamide gel electrophoresis SDS-PAGE). After electrophoresis, the gel was either directly stained with Coomassie Blue or subjected to Western blot analysis. For Western blot analysis the gel was blotted onto a nitrocellulose membrane and the membrane was incubated overnight with blocking buffer (phosphate-buffered saline (PBS) with 5% low-fat milk powder containing 0.05% Tween-20). After washing with PBST washing buffer (PBS/ 0.05% Tween-20), the membrane was incubated for 1 h with the anti-Htt antibodies MAB2166 (1:5000 diluted in PBST), 2B4 (1:1000 diluted in PBST) or anti-FLAG M2 antibody (1:1000 diluted in PBST), followed by an 1 h incubation with a peroxidase-coupled secondary antibody (Sigma), followed by detection with ECL detection reagent (Pierce) and autoradiography.

### Purification of full-length Htt proteins

Expression of Htt17 in B1.21 cells and of Htt46 in C2.6 cells was induced by adding Dox to the medium at a concentration of 1μg/ml. For expression of Htt17 and Htt128 following adenovirus-mediated in vitro gene transfer 293Cre66 cells were transduced with HC-Ad-Htt17Q-FH or with HC-Ad-Htt128Q-FH. Three days after induction with Dox or after vector-mediated transduction, respectively, the cells were harvested and lysed by freeze/thawing in lysis buffer (50 mM Tris, 500 mM NaCl, 5 mM EDTA, 5% glycerol and complete protease inhibitors (Roche)). The cell lysate was centrifuged at 30,000g for 3 hours and the supernatant was incubated with M2 anti-Flag beads (Sigma) (2 h, 4°C). Thereafter, the anti-Flag beads were washed in buffer A (50 mM Tris, 500 mM NaCl, 0.01% Tween 20, 5% glycerol, pH 8.0), buffer B (50 mM Tris, 500 mM KCl, 5 mM MgCl_2_, 0.01% Tween 20, 5% glycerol, pH 8.0), and buffer C (20 mM Tris, 200 mM KCl, 5 mM MgCl_2_, 5 mM ATP, 0.01% Tween 20, 5% glycerol, pH 8.0) and again buffer A. Htt protein was eluted with elution buffer (50 mM Tris, 500 mM NaCl, 0.01% Tween 20, 5% glycerol, 200 μg/ml FLAG peptide, pH 8.0). CHAPS and DTT were added to the eluate to a final concentration of 0.4% and 5 mM, respectively. The material was further purified by SEC using a Superose 6 10/300 GL column (GE Healthcare) in running buffer (20 mM Tris, 500 mM NaCl, 0.4% CHAPS, 5 mM DTT, 5% glycerol, pH 8.0). Htt eluted in two differently sized peaks. Htt fractions eluting in the major peak were combined and concentrated to about 200–500 μg/ml using Amicon ultra 30 kD filters (Millipore). The purity of Htt was estimated by SDS-PAGE of 2 μg of purified protein followed by Coomassie Blue staining.

### Sedimentation velocity (SV) analysis with AUC

Analytical Ultracentrifugation (AUC) was conducted with an Optima XL-A equipped with interference and absorbance optic. For sedimentation velocity experiments samples with 0.3 mg/mL concentration were filled in sapphire capped Epon double sector centerpieces with a path length of 12 mm. All samples were analysed in 20 mM Tris/HCl, 500 mM sodium chloride, 5mM DTT and 0.4% CHAPS, pH 8.0. Density and viscosity of the buffer were calculated with SEDNTERP [34]. Sedimentation was carried out at 42,000 rpm and 20°C in a four-whole Ti-60 Beckman Coulter rotor and monitored with interference optic and absorbance at 280 nm in continuous mode with 0.003 cm nominal radial increment, acquiring one replicate as scan. Data analysis was performed with Sedfit v. 14.1 from Peter Schuck [35], using a non-model based continuous Svedberg distribution method (c(S)). Ratios of different species were obtained by using the peak information tool of Sedfit.

### High resolution Native PAGE (HR-Native PAGE)

HR-Native PAGE was performed as described [36] with minor modifications. In brief, the native NuPAGE Novex Tris-Acetate gel system (Invitrogen) was used to perform electrophoresis under native conditions. Sodium cholate and dodecyl-β-D-maltoside (DDM) were added to the samples and to the cathode buffer to final concentrations of 0.1% and 0.04%, respectively, and proteins were separated in NuPAGE 3–8% Tris-Acetate gels using native Tris-Glycine buffer. Electrophoresis was performed at 150 V for about 4 h and the gel was stained with Coomassie Blue.

### CD spectroscopy

Before circular dichroism (CD) analysis of purified normal and mutant Htt a buffer exchange was performed (10 mM Potassium Phosphate, 50 mM Na_2_SO_4_, pH 7.4) using PD MiniTrap G25 columns (GE Healthcare). CD spectra from 190 nm to 240 nm were measured at 20°C on a JACSCO J-810 CD machine and 5 scans were averaged. The CD data were converted to mean residue ellipticity ([θ]) and analyzed with the CD spectra de-convolution program selcon3 at the CD analysis DichroWeb website [37].

### Size exclusion chromatography (SEC) for size and shape estimation of Htt

A Superdex-6 10/300 GL column (GE Healthcare) was calibrated with the gel filtration calibration kit HMW (GE Healthcare) and partition coefficient values (Kav values) of each standard protein were calculated using the equation Kav = (Ve-Vo)/(Vt-Vo) (Ve = elution volume for the protein, Vo = column void volume and Vt = total bed volume of the column). The calibration curve of Kav values versus the logarithm of molecular weights (MW) and the calibration curve of Kav values versus the logarithm of Stokes radius (Rs) were generated for the Superdex 6 10/300 GL column. Two hundred μl of Htt at a concentration of 200 μg/ml was applied to the column and Ve values of Htt proteins were determined from the mean of triplicate experiments. Kav values of Htt proteins were calculated and the MW and Rs of Htt proteins were calculated from calibration curves.

The friction ratio f/fmin and Htt MW were calculated as described by Erickson [38]. Rmin is the minimal radius of a sphere that could contain the given mass of protein. Rmin is determined by the equation Rmin = 0.066M^1/3^ (M is MW of protein in Daltons, Rmin in nanometer). Therefore, Rmin of Htt protein of 350 kD is calculated to be 4.63 nm. The friction ratio was determined by the equation f/fmin = Rs/Rmin (Rs is determined by SEC). The MW of Htt proteins was further determined by the equation M = 4205 (SRs) (S is in Svedberg units as determined by AUC, Rs in nm is determined by SEC and M is in Daltons) [38, 39].

### Htt protein analysis by mass spectrometry (MS)

For determination of phosphorylation and N-terminal acetylation of normal and mutant Htt, protein samples obtained following gene transfer with HC-Ad-Htt17Q-FH or with HC-Ad-Htt128Q-FH were purified as described above, except that phosphatase inhibitor (Thermo Scientific) at the recommended concentration was included during the purification procedure. Htt17 and Htt46 samples expressed in the Dox-inducible cell lines were also analyzed, however here without including phosphatase inhibitor during purification. For protein analysis by MS the recombinant proteins were digested in solution using trypsin according to in house protocols. Briefly, disulfide bridges were reduced using DTT and free thiols were alkylated with iodoacetamide. The protein solution was adjusted, if necessary, to pH 7.8 using ammonium bicarbonate. Proteins were digested using trypsin for 20 minutes at 37°C. The resulting peptides mixture was analysed using a nHPLC-MSMS (EasyLC-Orbitrap Velos) with a acetonitrile water system. Peptides were eluted in 60 minutes. Up to 15 tandem MS scans were allowed per spectral acquisition range.

For database searching, tandem mass spectra were extracted and charge states deconvoluted by Mascot Distiller version 2.2. Deisotyping was not performed. All MS/MS samples were analyzed using Mascot (Matrix Science, London, UK; version 2.2.06). Mascot was set up to search the ipi.HUMAN_FRCE.v3.87 database (3.87, 183784 entries) assuming the digestion enzyme trypsin. Mascot was searched with a fragment ion mass tolerance of 0.50 Da and a parent ion tolerance of 10.0 PPM. Iodoacetamide derivative of cysteine was specified in Mascot as a fixed modification. Oxidation of methionine, acetylation of the N-terminus and phosphorylation of serine, threonine and tyrosine were specified in Mascot as variable modifications.

Scaffold (version Scaffold_3.6.2, Proteome Software Inc., Portland, OR, USA) was used to validate MS/MS based peptide and protein identifications. Peptide identifications were accepted if they could be established at greater than 80.0% probability as specified by the Peptide Prophet algorithm [40]. Protein identifications were accepted, if they could be established at greater than 99.0% probability and contained at least 2 identified peptides. Protein probabilities were assigned by the Protein Prophet algorithm [41]. Proteins containing similar peptides and could not be differentiated based on MS/MS analysis alone were grouped to satisfy the principles of parsimony.

## Results

### Generation of two systems for expression of full-length normal and mutant Htt: doxycycline inducible cell lines and adenovirus-mediated gene transfer

In tissues or in immortalized cell lines endogenous Htt is expressed only at low levels and from these sources it would be very difficult to purify Htt at larger (mg) quantities, amounts that would be required for many biochemical or biophysical analyses. Therefore, for recombinant expression of full-length normal and mutant Htt we generated two expression systems, one based on stable cell lines and the other based on adenovirus mediated gene transfer.

For recombinant expression of Htt17 and Htt46 we generated the two cell lines B1.21 and C2.6, in which Htt expression is under control of the Tet-On inducible promoter system (Fig. 1A) [42]. In B1.21 cells expression of Htt17 and in C2.6 cells expression of Htt46 is induced upon addition of Dox to the cell culture medium. At 48 to 72 hours after induction the cells are harvested, at a time when significant intracellular cleavage of recombinantly expressed full-length Htt is not yet observed. Both versions of Htt were modified to contain a FLAG-His tag at the C-terminus of the protein to facilitate purification. The expression of the Htt proteins in both cell lines appeared to be quite strong allowing easy detection of Htt already upon Coomassie Blue staining following PAGE (Fig. 1C). Using purified full-length Htt as a standard we estimate that the cell-specific productivity in both cell lines for normal and mutant Htt is about 0.5 pg/cell following induction and harvesting at 48 to 72 hours (data not shown).

The isolation of suitable clones for expression of Htt with 17 or 46 Qs was rather inefficient. From more than 100 analyzed primary cell clones only a few cell lines were identified with high-level expression of full-length Htt following induction with Dox. From the best clones single-cell cloned were derived by limited dilution resulting in cell lines B1.21 (expressing Htt17) and C2.6 (expressing Htt46) with no (B1.21) or little (C2.6) leaky expression of recombinant Htt in the absence of Dox in the cell culture medium (Fig. 1C).

For vector-mediated expression of full-length normal and mutant Htt we generated high-capacity adenovirus (HC-Ad) vectors HC-Ad-Htt17-FH and HC-Ad-Htt128-FH (Fig. 1B), which carry the same Htt coding sequence and the C-terminal FLAG-His tag as were used for generation of the stable B1.21 and C2.6 cell lines described above. Different from ΔE1Ad vectors this vector type is characterized by a very low toxicity due to removal of all viral genes and a large cargo capacity of up to 35kb, thus allowing incorporation of the 11kb large expression cassettes coding for full-length normal and mutant Htt, respectively. For Htt production a human HEK 293 cell-based cell line was transduced and protein lysates were obtained 48 hours after transduction. Significant expression was observed already at the Coomassie Blue staining level with a visible band at the expected size (Fig. 1D) suggesting significant Htt expression levels.

The identity and integrity of the expressed full-length Htt versions (Htt17, 46, 128) were confirmed by Western blot analysis using anti-Htt and anti-FLAG antibodies. At the selected early harvest times, no or very little Htt degradation products were detected.

As expected, upon larger (> 72h) induction of B1.21 and C2.6 cells, also some degradation products, in addition to full-length Htt, were detected with anti-Htt and anti-FLAG antibodies (data not shown).

These data indicated that both the genetically engineered cell line and the gene transfer based system appeared to be suitable for production of full-length normal and mutant Htt.

### Establishment of a two-step procedure for efficient purification of full-length Htt from human cells

**Fig 2:**
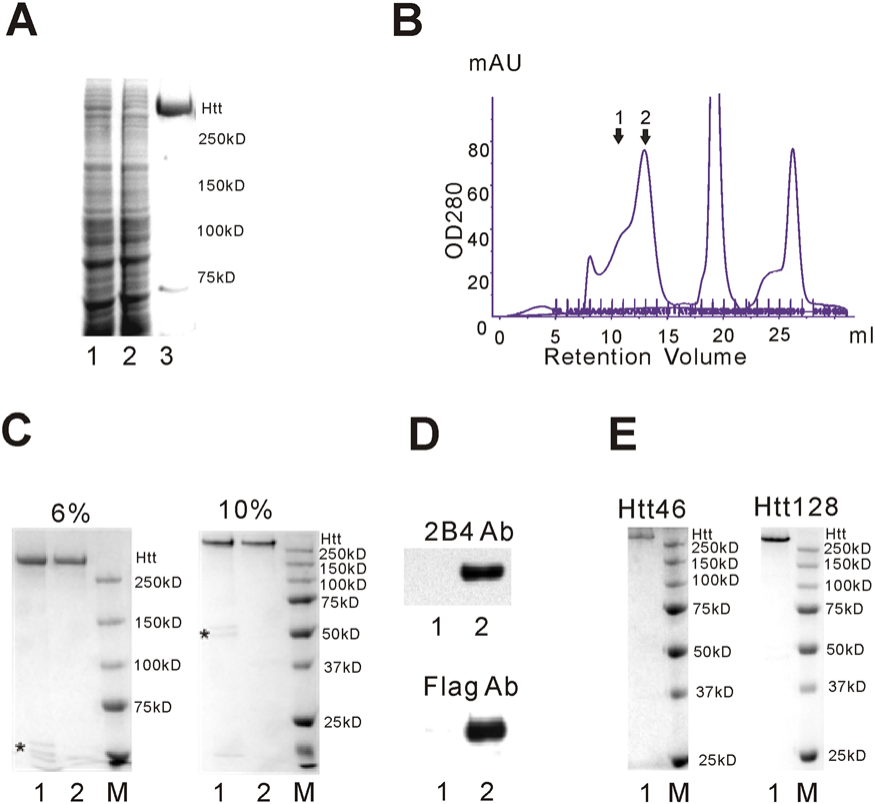
Htt purification using FLAG-affinity beads followed by size exclusion chromatography (SEC). (A) FLAG affinity purification of Htt17 from B1.21 cells. B1.21 cells were harvested 72 hours after induction and lysed by freeze/thawing. The cell lysate was cleared by centrifugation and filtration. Htt17 in the cleared cell lysate was purified by anti-FLAG beads. The cleared cell lysate (lane 1), the cell lysate after incubation with the anti-FLAG beads (lane 2) and Htt17 eluted from the anti-FLAG beads (lane 3) were analyzed by SDS-PAGE and Coomassie Blue staining. (B) Htt17, after elution from the anti-FLAG beads was further purified by SEC using a Superose 6 column in 20 mM Tris, 500 mM NaCl, 0.4% CHAPS, 5 mM DTT and 5% Glycerol. Htt eluted in a major peak [2] and a shoulder [1]. (C) Purified Htt17 was analyzed by SDS-PAGE and Coomassie Blue staining. 2 μg of FLAG-purified Htt (lane 1) and FLAG-SEC purified Htt (peak 2 from SEC, lane 2) was evaluated by 6% and 10% SDS-PAGE. Minor contaminating proteins in the FLAG-only purified Htt17 are indicated with an asterisk. Contaminating proteins were absent in FLAG-SEC purified Htt17. (D) The integrity of the FLAG-SEC purified Htt17 was confirmed by Western Blot analysis with the 2B4 anti-Htt antibody (reacting with the N-terminus of Htt) and Anti-FLAG antibody (detecting the C-terminus of Htt). Lane 1: unloaded; Lane 2: FLAG-SEC purified Htt17. (E) Analysis of Htt46 and Htt128 after FLAG-SEC purification. 2 μg FLAG-SEC purified Htt46 and Htt128 were evaluated by SDS-PAGE and Coomassie Blue staining. Contaminating proteins were not detected.

All recombinantly expressed full-length Htt versions contained a C-terminal FLAG tag that formed the basis for a two-step procedure to obtain Htt at high purity. Htt was nearly quantitatively depleted from the cell lysate by binding to FLAG affinity beads and was eluted by FLAG peptide using standard procedures (Fig. 2A) and only few contaminating proteins were visible upon staining with Coomassie Blue. While Htt was bound to the FLAG affinity beads, we introduced a washing step with ATP and MgCl_2_, since one of the contaminating proteins was found to be Hsp70 (determined by MS, data not shown) that, without the ATP/MgCl_2_ step, was not efficiently separated from Htt during the following size exclusion chromatography (SEC) (data not shown).

Following elution of Htt from the FLAG affinity beads by the FLAG peptide the protein was further purified by SEC using a Superose 6 column. Without inclusion of a detergent the protein apparently aggregated (data not shown). Following addition of CHAPS, DTT and increasing the salt concentration one major peak and one shoulder/minor peak containing Htt were observed in the chromatogram (Fig. 2B). The major peak (peak 2) contained Htt at high purity, mainly as a monomer while the shoulder/minor peak (labeled 1) mainly contained Htt oligomers (see below).

FLAG-purified and FLAG-SEC purified Htt17 was evaluated by analyzing 2 μg Htt17 by SDS-PAGE and Coomassie Blue staining (Fig. 2C).

Proteins of smaller size were removed by SEC and the purity of the final product was estimated to be at least 95% pure. The typical yield from 1x10^9^ B1.21 cells is about 1 mg Htt17 protein after FLAG affinity purification and about 0.5 mg Htt17 after FLAG-SEC purification.

By MS the bands running at smaller MWs in FLAG-purified Htt were identified as tubulins (data not shown). The identity and integrity of the FLAG-SEC purified Htt17 were further confirmed by Western blot analyses using anti-Htt and anti-Flag antibodies (Fig. 2D). Mutant Htt46 and Htt128 were also purified to the same degree as Htt17 by the same procedure (Fig. 2E).

### Detection of known and novel phosphorylation sites in normal and mutant Htt

Phosphorylation very likely plays important roles in regulating the functions(s) of Htt and for the toxicity in the case of polyQ expansion [22, 43–51]. Posttranslational modification of Htt17, Htt46 and Htt128 was analysed by Orbitrap LC/MS. Htt17 and Htt128 were analyzed following HC-Ad vector mediated gene transfer in 116 cells and Htt17 and Htt46 were analyzed from B1.21 and C2.6 cell derived material. All the cell types used for Htt expression are based on HEK293 cells, which are cells with a neuronal differentiation phenotype [52].

Overall sequence coverage of Htt17 and Htt128 produced following HC-Ad mediated gene transfer was 57% and 54%, respectively (S1 Fig.). All N-terminal peptides detected lacked the methionine and the next amino acid, alanine, was consistently acetylated. In Htt17 a total of 19 phosphorylated amino acids (serine, threonine and tyrosine) were detected, in Htt128 a total of 13 phosphorylation sites identified (Table 1).

**Table 1:** Phosphorylation sites identified by mass spectrometry from Htt purified from the HC-Ad vector transduced cells.

Residue Htt23Q	Htt17			Htt128		
	Observed proteolytic phosphopeptide	Mascot ion score	Mascot identity score	Observed proteolytic phosphopeptide	Mascot ion score	Mascot identity score
**T3**	atLEKLMK	29.1	35	atLEKLMK	30.4	34.8
**S419**	sGSIVELIAGGGSScSPVLSR	95.6	36.4	sGSIVELIAGGGSScSPVLSR	88.6	36.5
**S421**	SGsIVELIAGGGSScSPVLSRK	44.5	35.6	SGsIVELIAGGGSScSPVLSRK	64.2	35.7
**S431**	SRSGsIVELIAGGGsScSPVLSR	58.1	36.9	SRSGsIVELIAGGGsScSPVLSR	47.6	36.9
**S432**	SGSIVELIAGGGSscSPVLSRK	31.4	35.8	SGsIVELIAGGGSscSPVLSR	48	37.1
**S434**	SGSIVELIAGGGSScsPVLSR	86.5	36.4	SGSIVELIAGGGSScsPVLSR	62.9	36.4
**S642**	NMSHcRQPsDSSVDK	26.6	34.7			
**S1181**	AALPSLTNPPSLsPIRR	30.4	34.8	AALPSLTNPPSLsPIRR	28.1	32.2
**S1197**	EKEPGEQAsVPLSPK	47.5	36.1			
**S1201**	EKEPGEQASVPLsPK	40.5	36.1			
**S1351** ^**a**^	LGSSsVRPGLYHYcFMAPYTHFTQALADASLR	58.5	36.2	LGSSsVRPGLYHYcFMAPYTHFTQALADASLR	38.9	36
**Y1357** ^**a**^	LGSSSVRPGLyHYcFmAPYTHFTALADASLR	45.1	36.3			
**S1866**	HSLsSTKLLSPQmSGEEEDSDLAAK	72.9	37	HSLsSTKLLSPQmSGEEEDSDLAAK	34.6	36.9
**T1868** ^**a**^	HSLSStKLLSPQmSGEEEDSDLAAK	31.1	37			
**S1872**	HSLSSTKLLsPQMsGEEEDSDLAAK	29.5	36.7			
**S1876**	HSLSSTKLLsPQMsGEEEDSDLAAK	29.5	36.7	HSLSSTKLLSPQMsGEEEDSDLAAK	71.6	36.7
**T2337** ^**a**^	TNtPKAISEEEEEVDPNTQNPK	70.9	36.8	TNtPKAISEEEEEVDPNTQNPK	40.2	36.7
**S2653**	LGQVSIHSVWLGNSITPLREEEWDEEEEEEADAPAPSsPPtSPVNSR	28	35.5	LGQVSIHSVWLGNSITPLREEEWDEEEEEEADAPAPSsPPTSPVNSR	27.8	35.1
**T2656**	LGQVSIHSVWLGNSITPLREEEWDEEEEEEADAPAPSsPPtSPVNSR	28	35.5	LGQVSIHSVWLGNSITPLREEEWDEEEEEEADAPAPSsPPtSPVNSR	30.3	35.4

Htt23Q is used as the reference protein.

^a^ New phosphorylated sites identified in this study.

In addition to confirming the previously described phosphorylation of T3, S419, S421, S431, S432, S434, S642, S1181, S1197, S1201, S1866, S1872, S1876, S2653 and T2656 [43–47, 50, 53–59], additional phosphorylated sites were detected at S1351, Y1357, T1868 and T2337. In this analysis, some of the phosphorylation sites detected in Htt17 were not detected in Htt128 (Table 1), although this may have been a matter of sensitivity. As expected, all peptides from tryptic digests found to contain phosphorylated amino acids were also detected as unphosphorylated species.

In both Htt17 and Htt128 phosphorylation of the threonine in position 3 (T3) was found in a proportion of the peptides. The level of T3 phosphorylation in the two samples was comparable based on the extracted ion chromatogram (data not shown). In both samples serine 13 (S13) containing peptides were only found as unphosphorylated peptides, while S16 was not covered and thus could not be evaluated with respect to phosphorylation.

Htt17 and Htt46, purified from the Dox-inducible cell lines (however, purified in the absence of a phosphatase inhibitor), were analysed in an identical way. Coverage of Htt17 was 72% and of Htt46 69% (S2 Fig.) and detected phosphorylation sites of Htt17 and Htt46 are shown in Table 2. Previously not described phosphorylation sites were detected at S644, S1350, T1868 and S2550.

**Fig 3:**
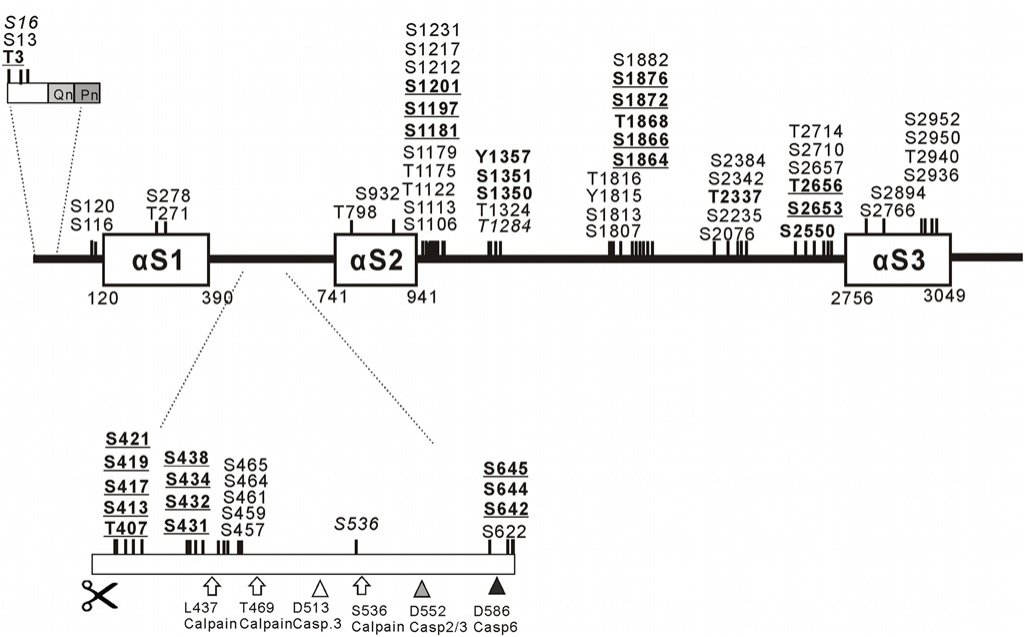
Schematic representation of Htt phosphorylation sites. Alpha-solenoid/Alpha-rod regions (αS1, αS2 and αS3) in Htt were predicted by the neural network-based ARD2 application in its most recent update ([60], http://cbdm.mdc-berlin.de/~ard2) for predicting HEAT repeats and related sequences. The phosphorylation sites were indicated with respect to the predicted alpha-solenoid domain structure of Htt. Phosphorylated amino acids detected in this study and not previously described are indicated in bold. Phosphorylated amino acids detected in this study and confirming previously described phosphorylation are indicated as bold/underlined. Previously described phosphorylation sites not covered by this study are indicated in italic. Previously described phosphorylation sites covered but not found phosphorylated in this study are indicated in regular letters. Known calpain cleavage sites (arrows) and known caspase 2/3, caspase 3 and caspase 6 cleavage sites (triangles) between alpha-solenoid regions 1 and 2 are indicated. As can be seen is this scheme, the phosphorylation sites mainly cluster in regions outside of predicted alpha-solenoid domains.

An overview of phosphorylation sites described previously and in this study is provided in Fig. 3. Tandem mass spectra from peptides with new phosphorylation sites are shown in S3–S9 Figs.

**Table 2:** Phosphorylation sites identified by mass spectrometry from Htt purified from the dox-inducible cell lines.

ResidueHtt23Q	Htt17			Htt46		
	Observed proteolytic phosphopeptide	Mascot ion score	Mascot identity score	Observed proteolytic phosphopeptide	Mascot ion score	Mascot identity score
**T3**				atLEKLMK	45.5	36.4
**T407**	TPPPELLQTLTAVGGIGQLtAAKEESGGRSR	43.4	42.6			
**S413**	TAAKEEsGGRSRSGSIVEL	39.4	41.7			
**S417**	TPPPELLQTLTAVGGIGQLTAAKEESGGRsR	39.3	42.5	TPPPELLQTLTAVGGIGQLTAAKEESGGRsR	52.4	38.3
**S419**				sGSIVELIAGGGSScsPVLSRK	46,9	40,2
**S421**	TAVGGIGQLTAAKEESGGRSRSGsIVEL	43.3	40.4	TAVGGIGQLTAAKEESGGRSRSGsIVEL	37.7	40.4
**S431**	SGSIVELIAGGGsScSPVLSR	64.3	43.3	SRSGsIVELIAGGGsScSPVLSR	52.4	40.2
**S432**	SGSIVELIAGGGSscSPVLSRK	99.6	39.5	SGSIVELIAGGGSscSPVLSR	42.7	39.8
**S434**	SRSGSIVELIAGGGSScsPVLSRK	63.9	43.5	SRSGSIVELIAGGGSScsPVLSR	81.6	39.9
**S438**	SGSIVELIAGGGSScSPVLsRK	61.2	39.6	sRSGSIVELIAGGGSScSPVLsR	46.9	40.3
**S642**	KNMSHcRQPsDSSVDKF	44.9	41.8	KNMSHcRQPsDSSVDKF	40.1	41.8
**S644** ^**a**^				NMSHcRQPSDsSVDKFVLRDEATEPGDQENKPcR	56.6	41.9
**S645**	NmSHcRQPsDSsVDKFVLRDEATEPGDQENKPcR	34	41.4	NMSHcRQPsDSsVDKFVLRDEATEPGDQENKPcR	47.8	41.4
**S1181**	AALPSLTNPPSLsPIR	44.8	40.1	AALPSLTNPPSLsPIR	46.4	37.3
**S1197**	EKEPGEQAsVPLSPK	41.4	38			
**S1201**	GKEKEPGEQASVPLsPK	45.5	42.1	GKEKEPGEQASVPLsPK	58	38.9
**S1350** ^**a**^	LGSsSVRPGLYHYcFMAPYTHFTQALADASLR	64.7	41.8			
**S1864**	RHsLSSTKLLSPQmSGEEEDSDLAAK	40.3	41.1	RHsLSSTKLLSPQMSGEEEDSDLAAK	56.5	41.2
**S1866**	AEVQQTPKRHSLsSTKL	51.8	40.4	HSLsSTKLLSPQMSGEEEDSDLAAK	87.2	40.9
**T1868** ^**a**^	RHSLSStKLLSPQMSGEEEDSDLAAK	44.7	41.2	RHSLSStKLLSPQMSGEEEDSDLAAK	102	41.3
**S1876**	LLSPQMsGEEEDSDLAAK	122	38.8	LLSPQMsGEEEDSDLAAK	124	38.7
**S2550** ^**a**^	KLsIIR	36.1	33	KLsIIR	44.7	32.1

Htt23Q is used as reference protein.

^a^ New phosphorylated sites identified in this study.

### Increased oligomer formation of purified Htt correlates with polyQ expansion

**Fig 4:**
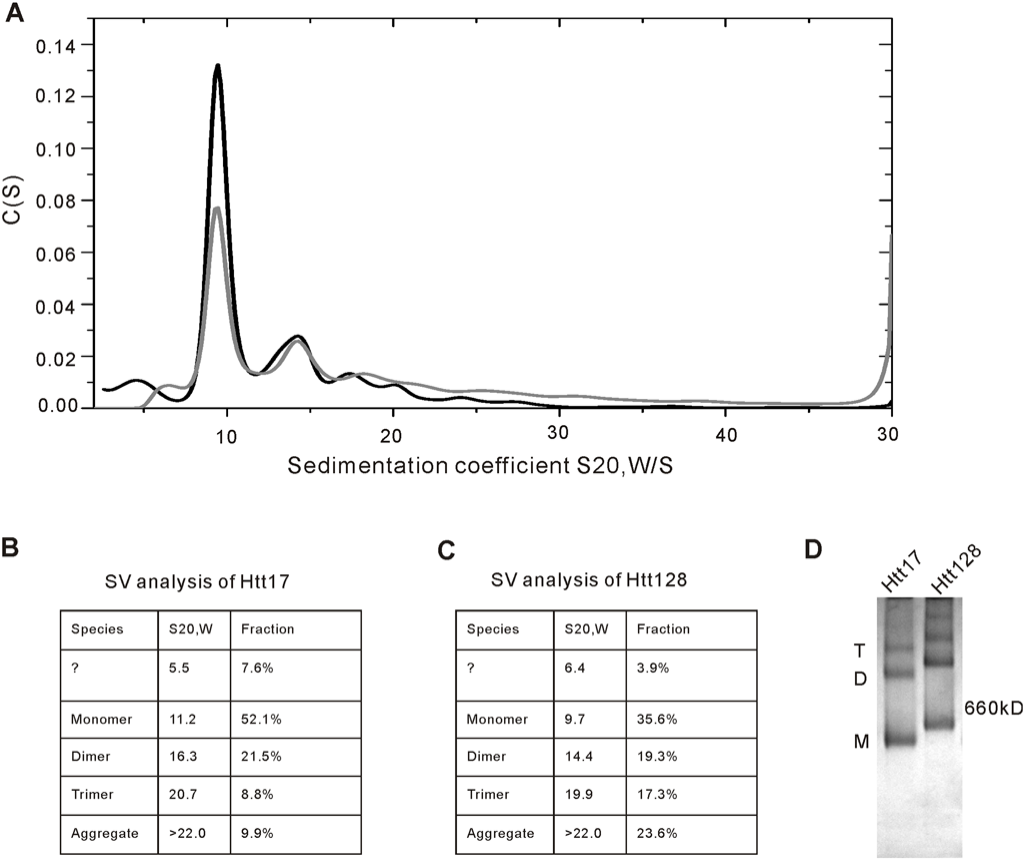
Polyglutamine expansion correlates with an enhanced formation of oligomers and aggregates. (A) c(s) distributions of Htt17 (black curve) and Htt128 (grey curve) obtained by Sedfit analysis, indicating one major peak at 10 S for the momoneric species. Dimers and trimers are observed around 15 S and 20 S, respectively. At s-values higher than 22 S oligomers of higher order and aggregates are detectable. (B, C) Summary of the sedimentation viscosity (SV) analysis of Htt17 and Htt128. The main species in both samples is the monomeric form (52.1% and 35.6%). Dimers have a fraction size from around 20% and trimers are higher populated in the Htt128 sample (17.3% vs. 8.8%). The amount of visible higher oligomers and aggregates is more pronounced in Htt128. Note: the AUC analyses of Htt17 and Htt128 were performed in two independent analyses likely explaining the smaller s_20,W_-value of Htt128 in this experiment. (D) Htt17 and Htt128 were analyzed with HR-native PAGE and Coomassie Blue staining with a result that was consistent with the SV analysis. M: Htt monomer; D: Htt dimer; T: Htt trimer.

To analyse the oligomeric status of Htt17 and Htt128, analytical ultracentrifugation was performed. Fig. 4A shows the c(s) distributions from Htt17 (black curve) and Htt128 (grey curve). The monomeric species has an s-value of 11.2 S for Htt17 and 9.7 S for Htt128 and is the main species of both proteins. Dimers and most likely trimers are detectable at 16.3 S and 20.7 S or 14.4 S and 19.9 S, respectively. In both cases additional aggregates are visible at sedimentation coefficients above 22 S. For Htt128 the amount of aggregates is two times more than for Htt17. Also the amount of the trimeric species is more populated than in the Htt17 sample. According to the Sedfit analysis the frictional ratios of Htt17 is 1.63 and for Htt128 1.60, which are in good agreement with the values detected in the later SEC analysis. The smaller species at 5.5 S and 6.4 S for Htt17 and Htt128 labeled with a question mark in Fig. 4B and 4C are putative degradation products.

We further analyzed formation of oligomers by high-resolution native PAGE (HR-native PAGE) (Fig. 4D), since upon regular native PAGE analysis only a smear and no distinct bands were observed, probably due to the hydrophobicity of Htt. In HR-native PAGE distinct bands were observed likely corresponding to monomer, dimer and trimer species as observed by AUC (see above). As also was observed by AUC, in Htt128 fewer monomers were observed and more oligomeric forms than in Htt17.

### Secondary structure determination by CD spectrum analysis

**Fig 5:**
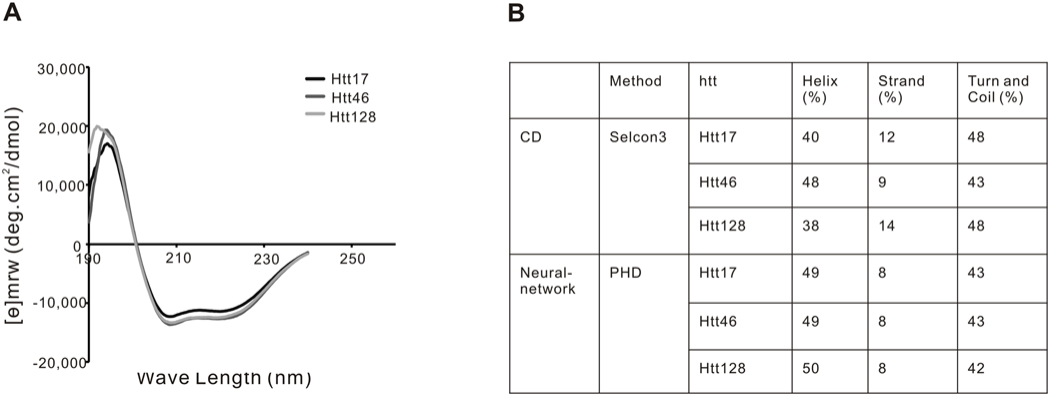
CD analysis of Htt17, Htt46 and Htt128. (A) CD spectra of Htt17, Htt46 and Htt128. (B) Based on the determined CD spectra, the secondary structures of Htt17, Htt46 and Htt128 were analyzed with Selcon3. Htt is not a purely helical protein (helix >50% and strand <5%), but rather a helix-rich protein (40% helix) mixed with strand (10%), which is consistent with the results of Htt secondary prediction by the PHD neuro-network. Significant differences in the secondary structure of Htt17, Htt46 and Htt128 were not observed.

CD spectra of full-length Htt17, Htt46 and Htt128 were obtained for secondary structure analysis. The CD spectra of the different Htt species have the typical characteristics of α-helical proteins with negative values at 208 nm and 222 nm and a positive peak value at 195 nm (Fig. 5A). Deconvolution of the CD spectra confirmed that the normal Htt17 is composed of about 40% α-helix, 12% β-strand and 48% turn and coil, which is in agreement with the Neural Network secondary prediction [61] of 49% α-helix, 8% β-strand and 43% turn and coil (Fig. 5B). Clear differences in the CD spectra between Htt17, Htt46 and Htt128 were not observed.

### Htt is a protein with an elongated shape as determined by analytical size exclusion chromatography (SEC)

**Fig 6:**
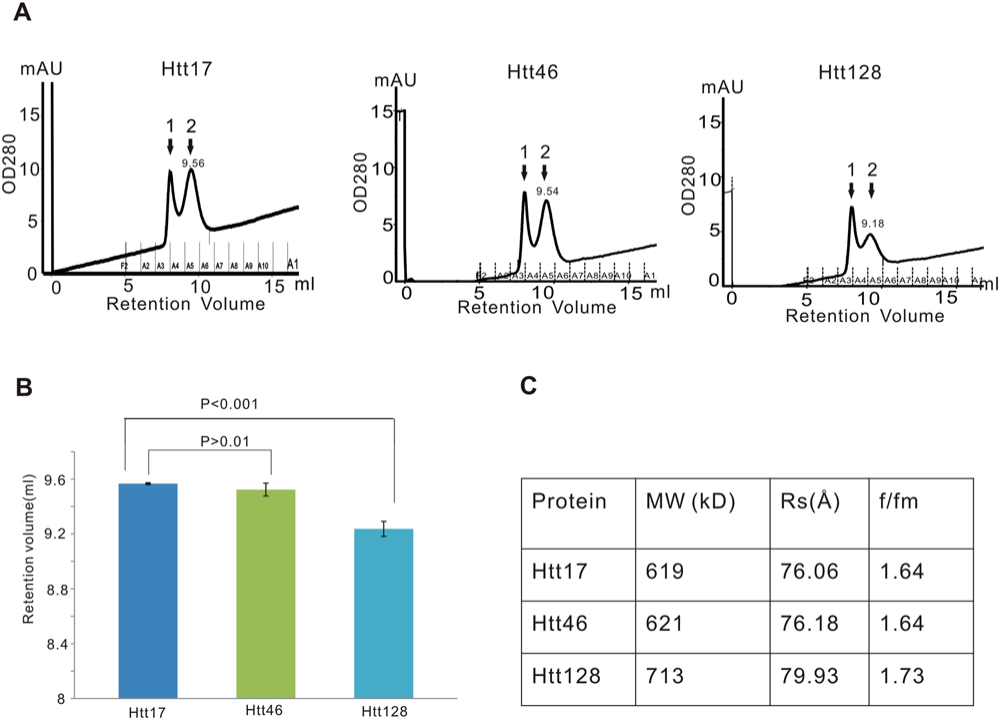
Analytical size exclusion chromatography. Htt is a molecule with elongated shape and mutant Htt128 is more extended than Htt17 and Htt46. (A) Htt17, Htt46 and Htt128 were analyzed by SEC with a Superdex-200 10/300 GL column to estimate the molecular weight (MW) and stoke radius (Rs). Htt oligomers eluted at peak 1 corresponding to the void volume of this column and Htt monomers eluted at peak 2. Htt17 and Htt46 eluted at retention volume of about 9.5 ml, while Htt128 eluted at a retention volume of about 9.18 ml. (B) There were no significant differences of the retention volumes between Htt16 and Htt46, while Htt128 eluted significantly earlier than Htt17 and Htt46. (C) From the SEC results and considering a calibration curve the MW of Htt would be calculated to have a MW of about 600 kD, which is significantly larger than its actual 350 kD MW, indicating that Htt is not a globular protein. The friction ratio f/fm represents the total shape asymmetry of molecules. The f/fm of 1.6 predicts that Htt has an elongated shape. The larger f/fm value of Htt128 than Htt17 and Htt46 indicated that the shape of Htt128 was more extended than the shapes of Htt17 and Htt46.

Htt17, Htt46 and Htt128 were analyzed by SEC using a Superdex 200 column to estimate size and shape of Htt protein and any potential effect of the polyQ expansion on protein conformation. Htt17 and Htt46 eluted at a retention volume of about 9.5 ml, while Htt128 eluted at a retention volume of about 9.18 ml, thus significantly earlier than Htt17 and Htt46, suggesting that the polyQ expansion in Htt128 might affect the conformation of Htt (Fig. 6A and 6B). Proportions of Htt17, Htt46 and Htt128 also eluted in the void volume (Fig. 6A). Since Superdex 200 has a separation range for molecules between 10 kD and 600 kD, monomers but not oligomers of Htt would be expected to elute within the separation range of this column while Htt oligomers should elute in the void volume. Therefore the result confirmed our AUC results that Htt proteins also may interact with each other to form protein oligomers. The elution volume of Htt128 monomers was much less than of Htt17 and Htt46 monomers, which again indicated that the extended polyQ in Htt128 increased the propensity of Htt proteins to form Htt oligomers and aggregates. The elution patterns of Htt17 and Htt46 were identical and the moderate polyQ expansion in Htt46 apparently did not affect its running behavior.

From the elution pattern (Fig. 6C) and the calibration curve, set up with globular proteins, we derived an indication of the shape of the different Htt species: Htt17 runs at a molecular weight (MW) of 600 kD, significantly larger than its actual MW of 350 kD, indicating that Htt17 is not a globular protein.

Upon SEC, proteins do not separate according to their MW but according to their frictional coefficient (f) or Stokes radius (Rs) [39]. Rs is defined as the radius of a smooth sphere that diffuses at the same rate as the molecule or would have the actual f of the molecule, which is determined by both the asymmetry and hydration of the molecule. Rs is smaller than the effective radius and an extended protein would have a larger Rs than a globular protein of the same actual MW. We calculated Htt17 to have a Rs of 76,06 Å, which is even larger than the Rs of ferritin, a 450 kD globular protein with a Rs of 61Å, further indicating that Htt17 is a protein with a rather extended conformation. After surveying of f/fmin ratios for a variety of protein of known structure, Erickson [38] proposed that the friction ratio f/fmin is an excellent predictor of the asymmetry of a protein: a typical globular protein has a f/fmin ratio between 1.2 and 1.3; a moderately elongated protein has a f/fmin in the range of 1.5–1.9 and a highly elongated protein like fibrinogen has a f/fmin in the range of 2.0–3.0 [38]. Based on the Rs, Htt17 is calculated to have a f/fmin of 1.64 consistent with the AUC data, suggesting that Htt17 is a protein with a moderately elongated shape. Based on the sedimentation coefficient (S) of Htt17 obtained by the AUC results, we can calculate the MW of Htt17 by the Siegel and Monty equation ((M = 4205(SRs), S is in Svedberg unit, Rs is in nm and M in Dalton [38, 39]), resulting for Htt17 in a MW of 358 kD, which is consistent with its real MW based on sequence information. These data also confirmed that the eluted Htt17 is a Htt monomer. Based on our calculation Htt46 has a similar Rs and f/fmin, respectively, as Htt17 (76.18 Å to 76.06 Å, 1.64 to 1.64), while Htt128 has larger a Rs and and f/fmin ratio than Htt17 (79.93 Å to 76.06 Å, 1.73 to 1.64), indicating that Htt128 has a more elongated shape than both Htt17 and Htt46.

## Discussion

Scalable production of both normal and mutant full-length Htt would greatly facilitate biochemical and biophysical studies aiming to better understand the physiological function(s) of Htt and, in case of polyQ expansion, the pathogenesis of HD. Both production systems described in this report in principle allow for production of Htt in larger quantities than has been described before, when Baculovirus based expression in insect cells was performed [26, 27]. Differently from previous studies [26, 27], the affinity tag was placed to the C-terminus of Htt with the idea that a) the N-terminus of Htt would be more likely in its natural configuration and not influenced by an extra peptide used for purification, and b) purification of full-length Htt would be facilitated and at the same time the contamination with shorter Htt fragments, potentially generated by incomplete translation of the mRNA, be avoided. With respect to the Dox-inducible cellular system we have recently successfully adapted the different cell lines to suspension cell culture further facilitating production scale-up (unpublished data).

The HC-Ad vector based production system has the additional advantage that different cell types including neuronal cell types can be used for expression and production, a property that would be of interest when analyzing, for example, cell-specific posttranslational modification of Htt or when studying the interaction of Htt with other proteins.

Purification was based on a two-step protocol taking advantage of a C-terminal FLAG epitope for affinity purification followed by size exclusion chromatography. Crucial for purification and keeping the protein soluble appeared to be the presence of a detergent. A hydrophobic character of Htt has also been suggested by previous studies. Htt is associated with cellular membranes and vesicles [62–66] and has been found in the membrane fraction of the brain in proteomic studies [67]. Embryonic stem cells from homozygous Htt knockout mice and cells overexpressing Htt had defects in cellular membrane systems (ER, Golgi and recycling endosomes) [68]. Htt is involved in intracellular vesicular trafficking including exocytosis, endocytosis, axonal transport, endosomal motility and post-Golgi trafficking [69–72] and Htt has also been shown to directly interact with membranes: an N-terminal membrane-association signal has been found to confer the association of Htt with the ER, late endosomes and autophagosomes [73]; amino acids 172–372 of Htt mediate Htt association with acidic phospholipids at the plasma membrane [74] and palmitoylation of Htt influences its cellular localization [75]. The hydrophobicity of Htt supports the concept that Htt directly interacts with intracellular membranes via exposed hydrophobic regions and may be functional as a membrane-associated protein.

X-ray structure data of full-length Htt have not been reported. Based on structure prediction programs it is likely that Htt folds in a structure consisting of several compact HEAT repeat domains and of areas of disorder as they are found in intrinsically disorders proteins (IDPs) (reviewed in [76]). HEAT repeats consist of anti-parallel alpha helices that are connected by a short linker in a helix-turn-helix format and that are stacked to form higher-order molecular structures, designated as solenoids or as alpha-rods [77]. HEAT repeat-rich proteins with known three-dimensional structures such as importin-β, karyopherin-β2, the PR65/A subunit of PP2A and Cand1 [78–81] are almost completely composed of HEAT repeats, have a very high (>70%) α-helical content and less than 2% β-strands. Our data from CD analyses indicated that Htt has a lower helical content (40–50%) than “classical” HEAT proteins and more β-strand structures (10%). In a previous CD study with Htt produced in insect cells similar findings were reported, although with a somewhat higher α-helix and lower β-strand content [26].

Interestingly, we did not detect any clear differences in the CD spectra of Htt17, Htt46 and Htt128 suggesting that the overall secondary structure of Htt was not significantly influenced by the mutation, at least not under the conditions used in this analysis.

Using sequence homology-based prediction programs different numbers of HEAT repeats have been grouped in either 3 or 4 clusters and aligned to the primary Htt sequence [25, 82, 83]. In a neural network-based alternative approach for prediction of HEAT repeats and related sequences [77] in a recently updated version (http://cbdm.mdc-berlin.de/~ard2) [60] three HEAT-repeat containing alpha-solenoids can be defined between amino acids 120 and 390, 741 and 941, and 2756 and 3049 ([77] and data not shown).

Analysis of tryptic digests of normal and mutant Htt by MS resulted in the detection of several phosphorylation sites that previously have not been described. As expected, most phosphorylations were found at serine and threonine positions, although in one instance a tyrosine was found phosphorylated (Y1357), to our knowledge only the second tyrosine modification in Htt described so far. In addition, many of the previously described phosphorylation sites, derived from a larger number of studies, were confirmed by this analysis. As can be seen in Fig. 3, nearly all detected phosphorylation sites locate outside of alpha-solenoid domains as identified by prediction with the ARD2 application ([60] and data not shown). Many locate to identified PEST domains [83, 84], regions enriched in proline (P), glutamic acid (E), serine (S) and threonine (S) and considered as targets for protease activity. We note that most detected phosphorylation sites also locate to disordered protein regions as predicted using the neural network based PONDR prediction program [76, 85, 86]. We consider this catalogue a snapshot of phosphorylation under specific cellular conditions, rather than a definitive overview, also since in both production systems Htt was significantly overexpressed. The amount of Htt produced per cell was about 0.5 pg, corresponding to close to 10^6^ molecules per cell. On the other hand, the fact that even under overexpression conditions many known phosphorylation sites were confirmed, gives significance to this data including to the newly described PTMs.

The N-terminus of Htt likely plays a number of roles in the function of Htt under normal and pathological conditions and it is known to influence the biophysical behavior of Htt in vitro and likely in vivo (reviewed in [76]). For example, while the N-terminal peptide alone resists aggregation, adding it to a polyQ results in strong aggregation [84]. Confirming previous results [50] obtained with a N-terminal Htt exon 1 protein fragment following transfection in ST14A cells (rat cell line] and HeLa cells (human cell line) we observed that in our analyses of full-length Htt all N-terminal peptides lacked the methionine and alanine as the next amino acid was consistently found acetylated. This in itself is not surprising since in 80–90% of proteins the amino acid following the methionine, if it is an A, S, V, T or C, is acetylated by the N-terminal acetyl transferase NatA, after removal of the methionine by methionine aminopeptidase [87]. However, it is conceivable that this modification may impact on some of N-terminal Htt's interactions, on posttranslational modifications that take place at the N-terminus or on the biophysical behavior of N-terminal fragments *in vitro* and/or *in vivo*. It would be interesting to analyze the Htt N-terminus with respect to this modification in primary tissues.

Another modification of significant interest has been the phosphorylation of threonine 3 (Thr-3). Direct determination in HD mouse models of Thr-3 phosphorylation of Htt in cortex versus striatum, use of phosphomimetic mutants in drosophila and *in vitro* studies together suggested that Thr-3 phosphorylation might be neuroprotective in the case of polyQ expansion, while leading to increased aggregate formation [50]. In our own studies performed in 293 cells and expressing full-length Htt either following adenovirus-mediated or Dox-inducible expression, Thr-3 phosphorylation was detected in Htt17 and Htt128, with comparable levels based on the extracted ion chromatogram.

We could also confirm two data sets from a previous study [26], obtained with Htt produced in insect cells, that purified Htt has an elongated shape and that it tends to form dimers and higher order oligomers. Analytical SEC (Fig. 6) indicated that the stokes radius was significantly larger than expected if Htt were a globular protein. While the stokes radius of Htt17 and Htt46 did not differ, the elution profile of Htt128 with a lower retention volume suggested a further alteration in its shape. Simulations of the frictional ratios with the program Sedphat on the basis of the AUC results confirm this implication (data not shown). However, in SEC both normal and mutant Htt were obtained mainly as monomers, significant dimer formation and also higher order oligomers were noted both by AUC and by HR-native PAGE (Fig. 4). Interestingly, in Htt128 and compared to Htt17 a lower amount of monomers (35.6% versus 52.1%), and a higher amount of trimers (17.3% versus 8.8%) and also of aggregates (23.6% versus 9.9%) were observed.

The reason for formation of dimers, trimers and higher order oligomers is currently not clear. We believe that most likely oligomers are formed during purification and/or concentration of the recombinantly produced Htt, at least to a large part. However, intracellular formation of Htt oligomers cannot be excluded, also since oligomer formation of full-length Htt has been observed even in material purified from human brain specimen [88]. We can only speculate about possible molecular mechanisms for the generation of full-length Htt oligomers. Most aggregation studies have been performed with polyQs of different length, either alone, linked to the first 17 amino acids of Htt (Htt^NT^) plus further c-terminal peptides such as the polyP in some studies. Interestingly, in one study, in which the biophysical behavior of Htt^NT^ linked to polyQ of different length was studied, it was observed that Htt^NT^, with or without polyQs added existed in as an equilibrium between disordered monomers, alpha-helical tetramers and higher order oligomers; when more than 8 glutamines were added, amyloid-like aggregates were formed. Could it be that the oligomers observed in our study and in insect cell produced material [26] are held together by the N-terminal peptide? Our observation of increased oligomer formation with mutant full-length Htt at least suggests that the N-terminal part of Htt might be involved in this process. Another possibility to potentially explain oligomer formation might reside in the known ability of HEAT domains to mediate protein-protein interaction. Neural network-based structure prediction of full-length Htt and experimental studies suggested both intramolecular interactions of alpha-solenoid/rod domains and also, via alpha-solenoid/rod 2 (see Fig. 3), interaction between two full-length Htt monomers [77].

In summary, we have developed two scalable processes for production of full-length normal and mutant Htt in human cell lines and have characterized the recombinant Htts side by side. Consistent with Htt purified from insect cells, Htt produced in human cells is a helix-rich protein that has an elongated rather than a globular shape. Purified Htt is a hydrophobic protein that tends to form dimers, trimers and higher order oligomers. The tendency to form oligomers and aggregates was increased with mutant Htt. We also present an overview on phosphorylation of both normal and mutant Htt produced in human cells, confirming many previously described phosphorylation sites and adding several new ones that overall mainly locate to areas between predicted alpha-solenoid domains. As a further post-translational modification the N-terminus of full-length Htt was found consistently acetylated.

The ability to produce normal and mutant full-length Htt in human cells at large scale should facilitate further biochemical and biophysical analysis of Htt and in the long run contribute to a better understanding of the pathogenesis in HD.

## Supporting Information

S1 Fig Peptide coverage of Htt, produced by HC-Ad vector mediated expression and analysed by MS. Detected peptides are indicated in yellow, modified amino acids in green. (A) Analysis of Htt17. (B) Analysis of Htt128.
(TIF)

S2 Fig Peptide coverage of Htt, produced by Dox-induced expression in cell lines and analysed by MS. Detected peptides are indicated in yellow, modified amino acids in green. (A) Analysis of Htt17. (B) Analysis of Htt46.
(TIF)

S3 Fig Example tandem mass spectrum of S644 phosphorylation from KNMSHCRQPSDSSVDKF. (TIF)

S4 Fig Example tandem mass spectrum of S1350 phosphorylation from LGSSSVRPGLYHYCFMAPYTHFTQALADASLR. (TIF)

S5 Fig Example tandem mass spectrum of S1351 phosphorylation from LGSSSVRPGLYHYCFMAPYTHFTQALADASLR. (TIF)

S6 Fig Example tandem mass spectrum of Y1357 phosphorylation from LGSSSVRPGLYHYCFMAPYTHFTQALADASLR. (TIF)

S7 Fig Example tandem mass spectrum of T1868 phosphorylation from HSLSSTKLLSPQMSGEEEDSDLAAK. (TIF)

S8 Fig Example tandem mass spectrum of T2337 phosphorylation from TNTPKAISEEEEEVDPNTQNPK. (TIF)

S9 Fig Example tandem mass spectrum of S2550 phosphorylation from KLSIIK. (TIF)
