# Supplementary figures and images for: Scalable Production in Human Cells and Biochemical Characterization of Full-Length Normal and Mutant Huntingtin
Source: PLoS One. 2015 Mar 23;10(3):e0121055. doi: 10.1371/journal.pone.0121055 (PMC4370734; doi:10.1371/journal.pone.0121055)

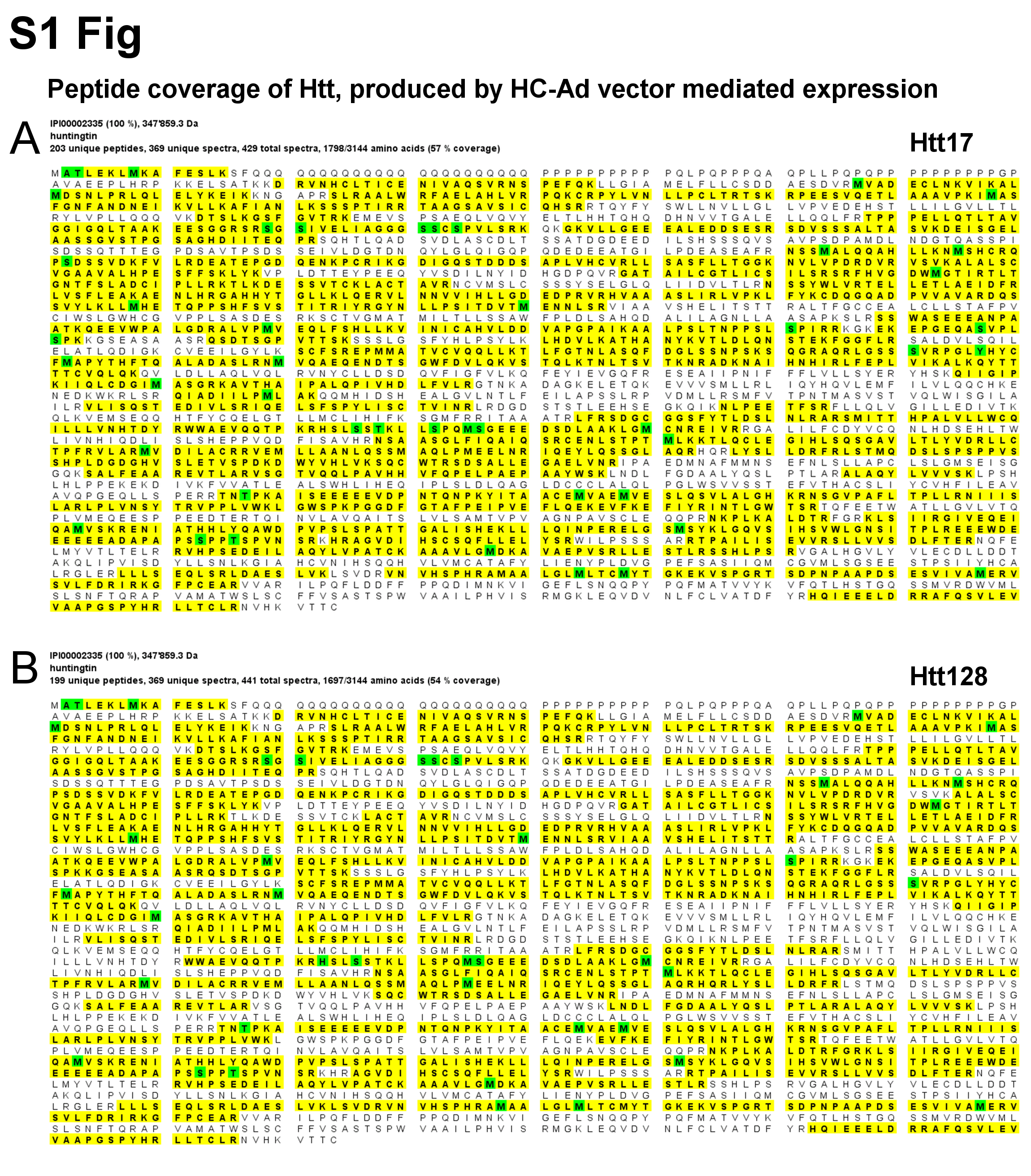

Supplement: S1 Fig — Detected peptides are indicated in yellow, modified amino acids in green. (A) Analysis of Htt17. (B) Analysis of Htt128. (TIF) [file pone.0121055.s001.tif]

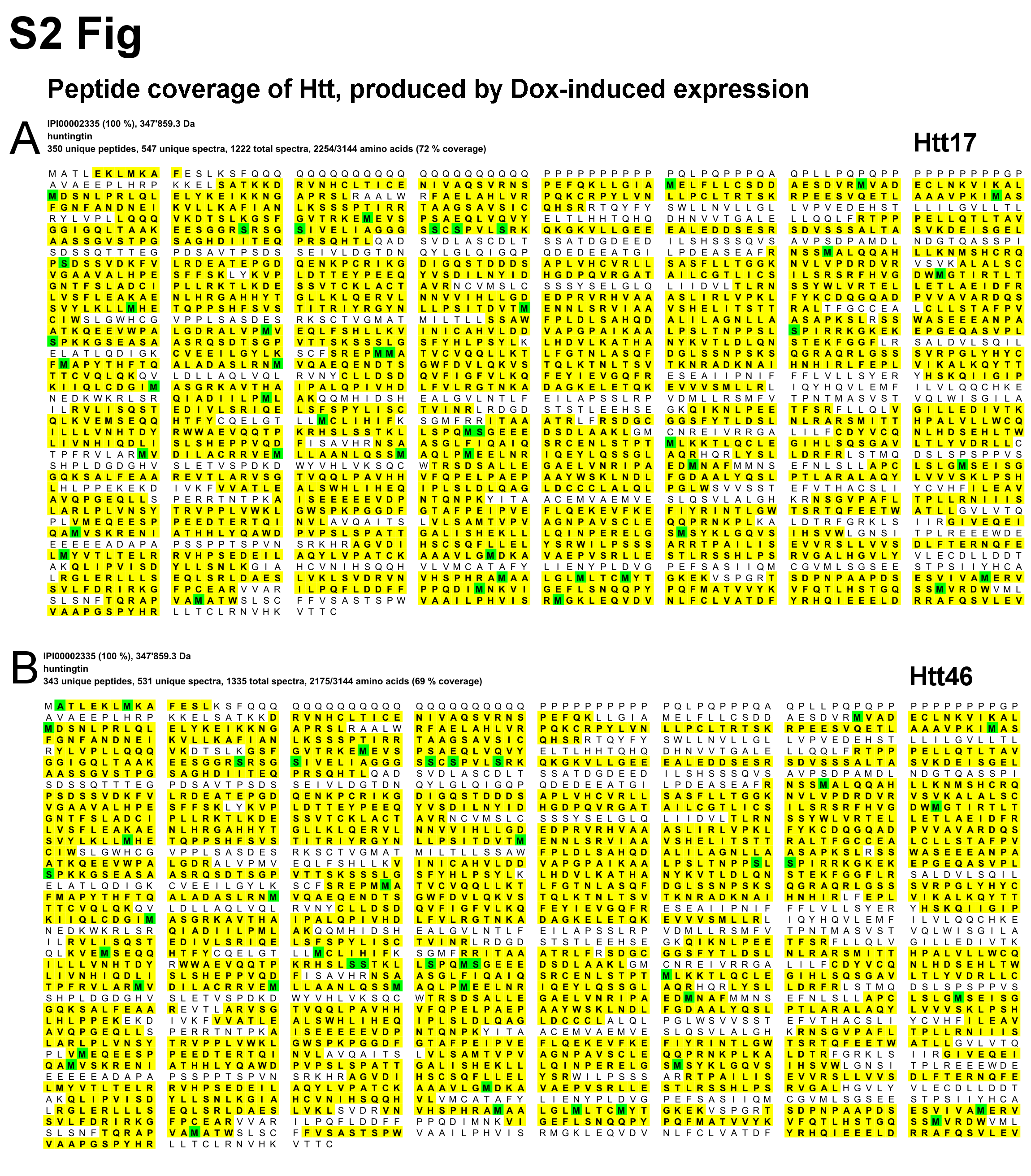

Supplement: S2 Fig — Detected peptides are indicated in yellow, modified amino acids in green. (A) Analysis of Htt17. (B) Analysis of Htt46. (TIF) [file pone.0121055.s002.tif]

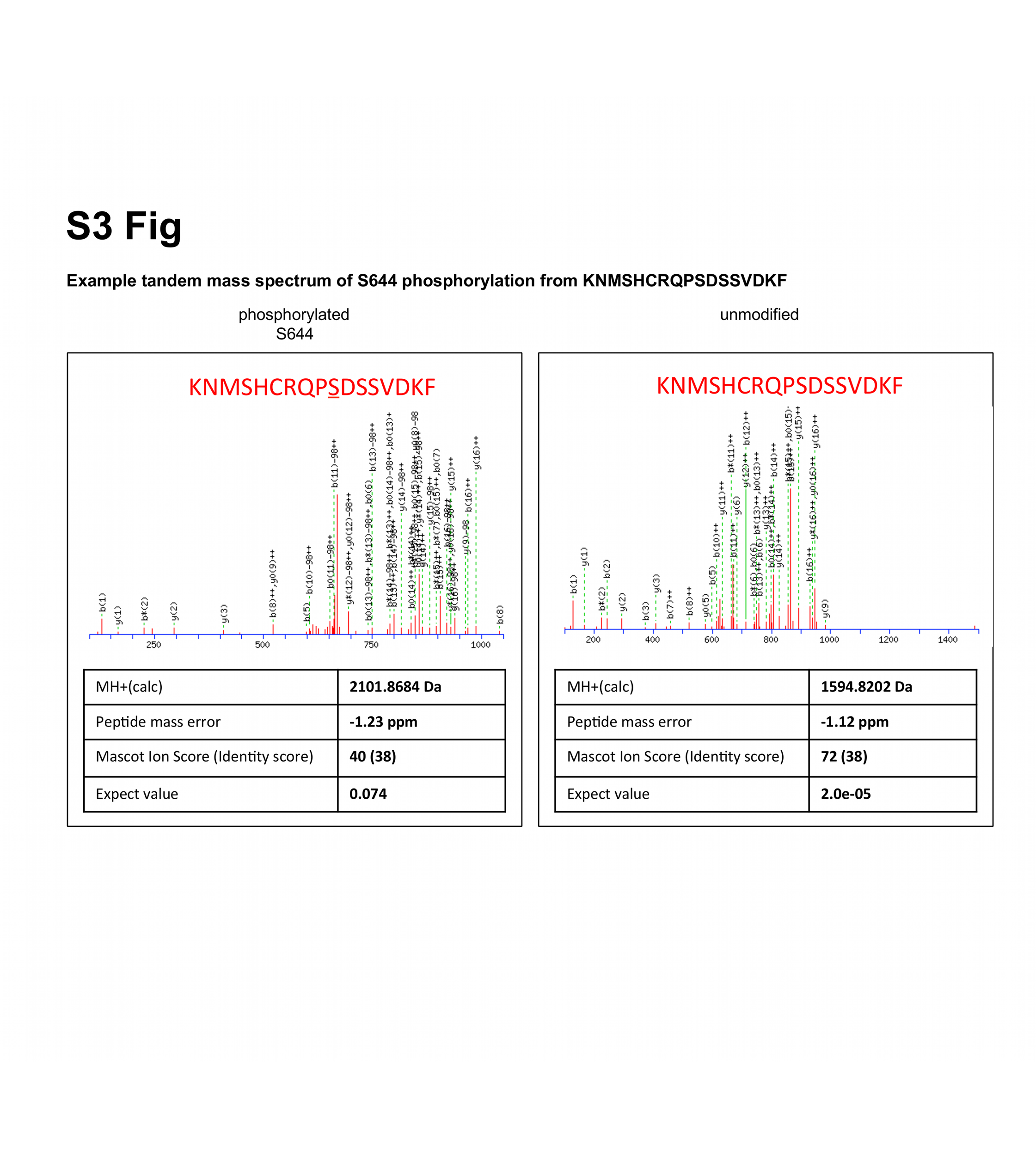

Supplement: S3 Fig — (TIF) [file pone.0121055.s003.tif]

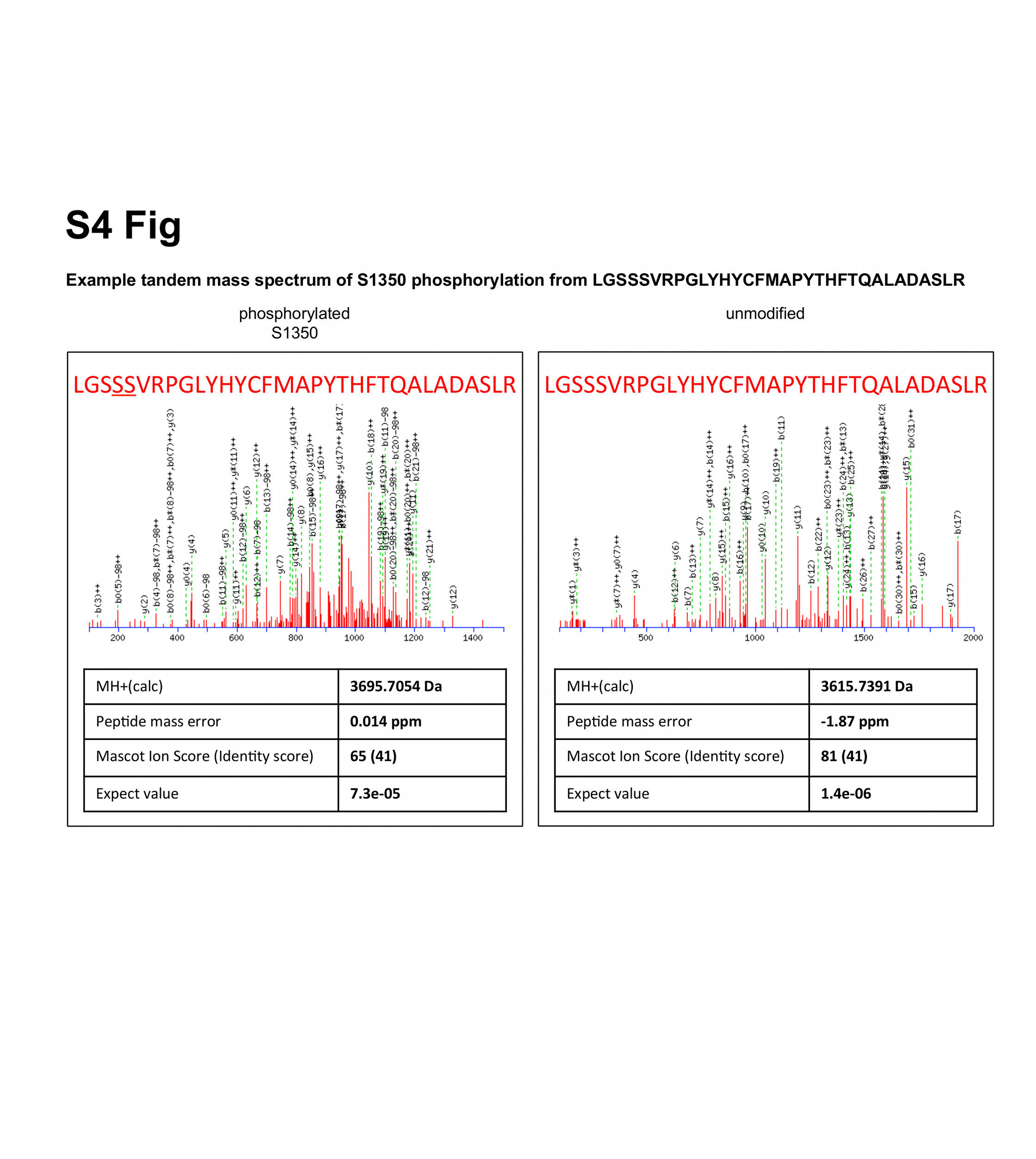

Supplement: S4 Fig — (TIF) [file pone.0121055.s004.tif]

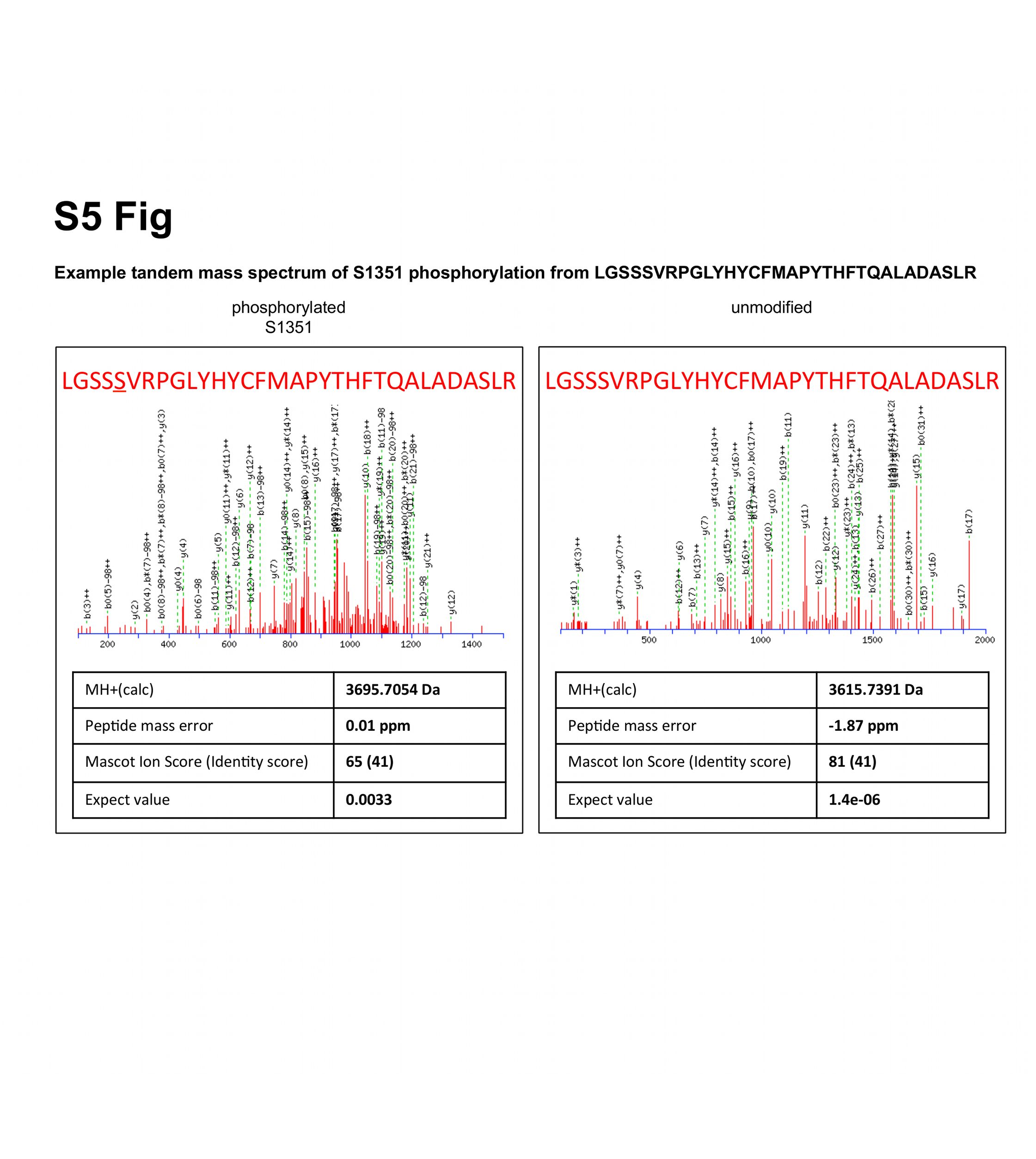

Supplement: S5 Fig — (TIF) [file pone.0121055.s005.tif]

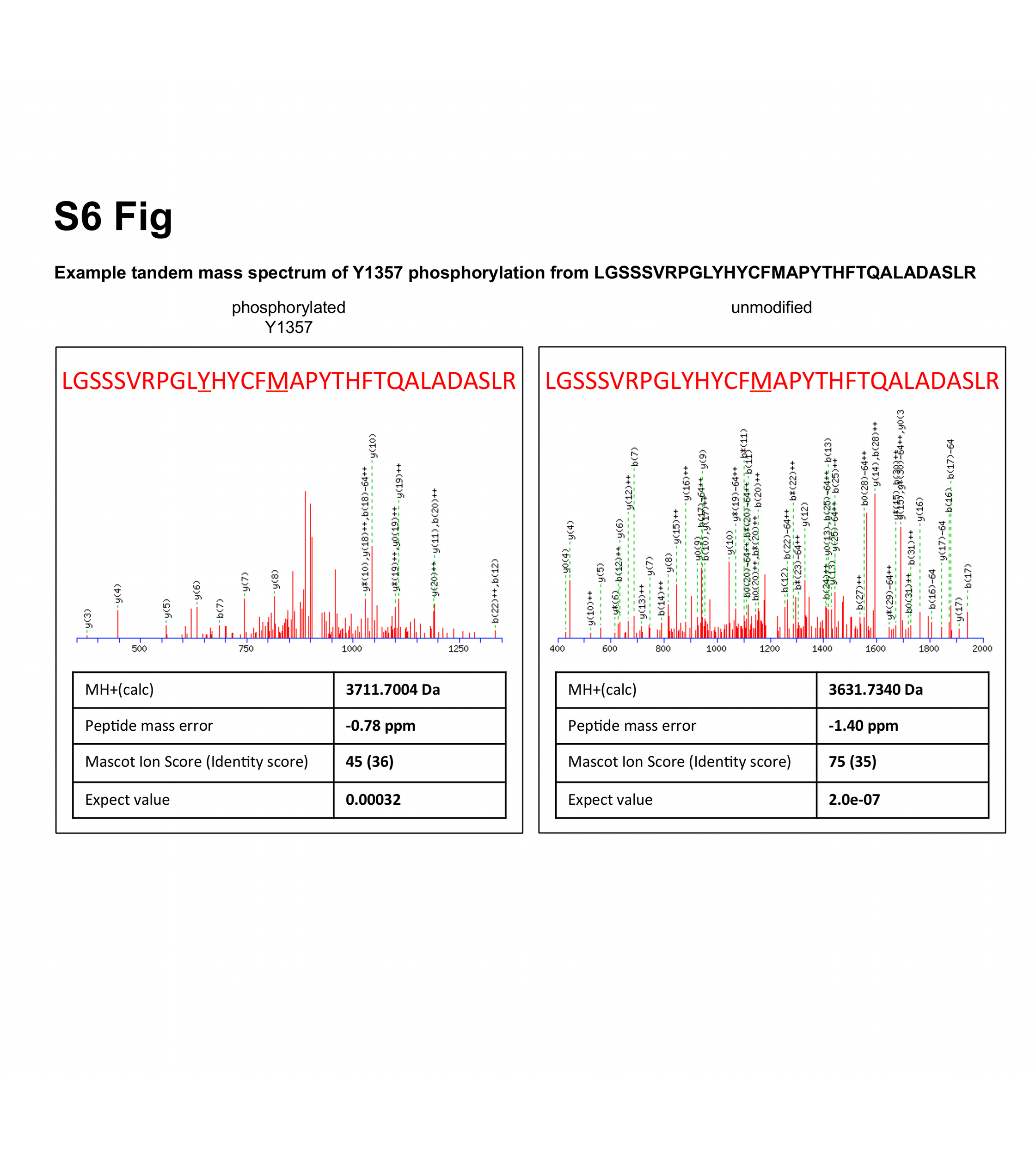

Supplement: S6 Fig — (TIF) [file pone.0121055.s006.tif]

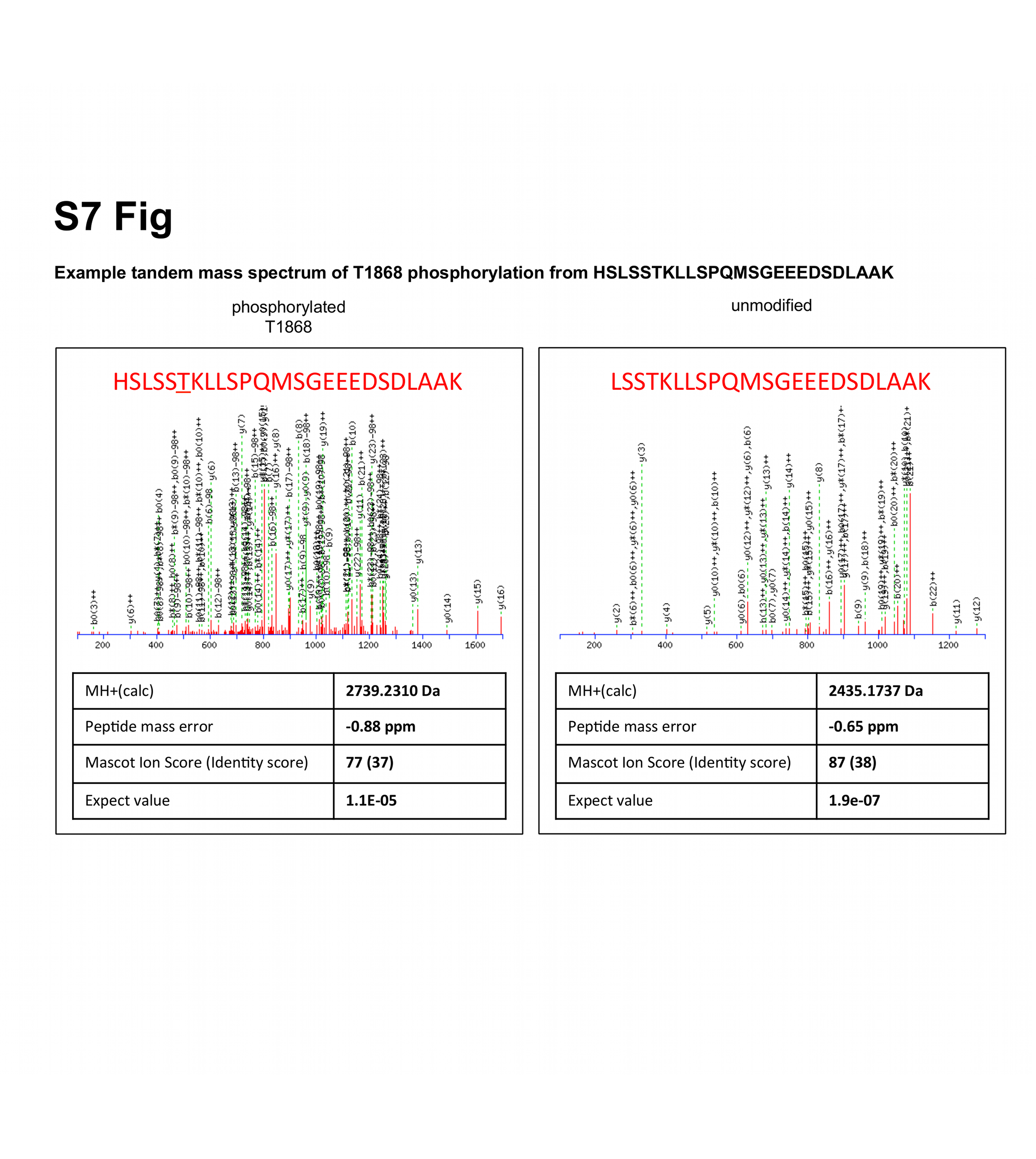

Supplement: S7 Fig — (TIF) [file pone.0121055.s007.tif]

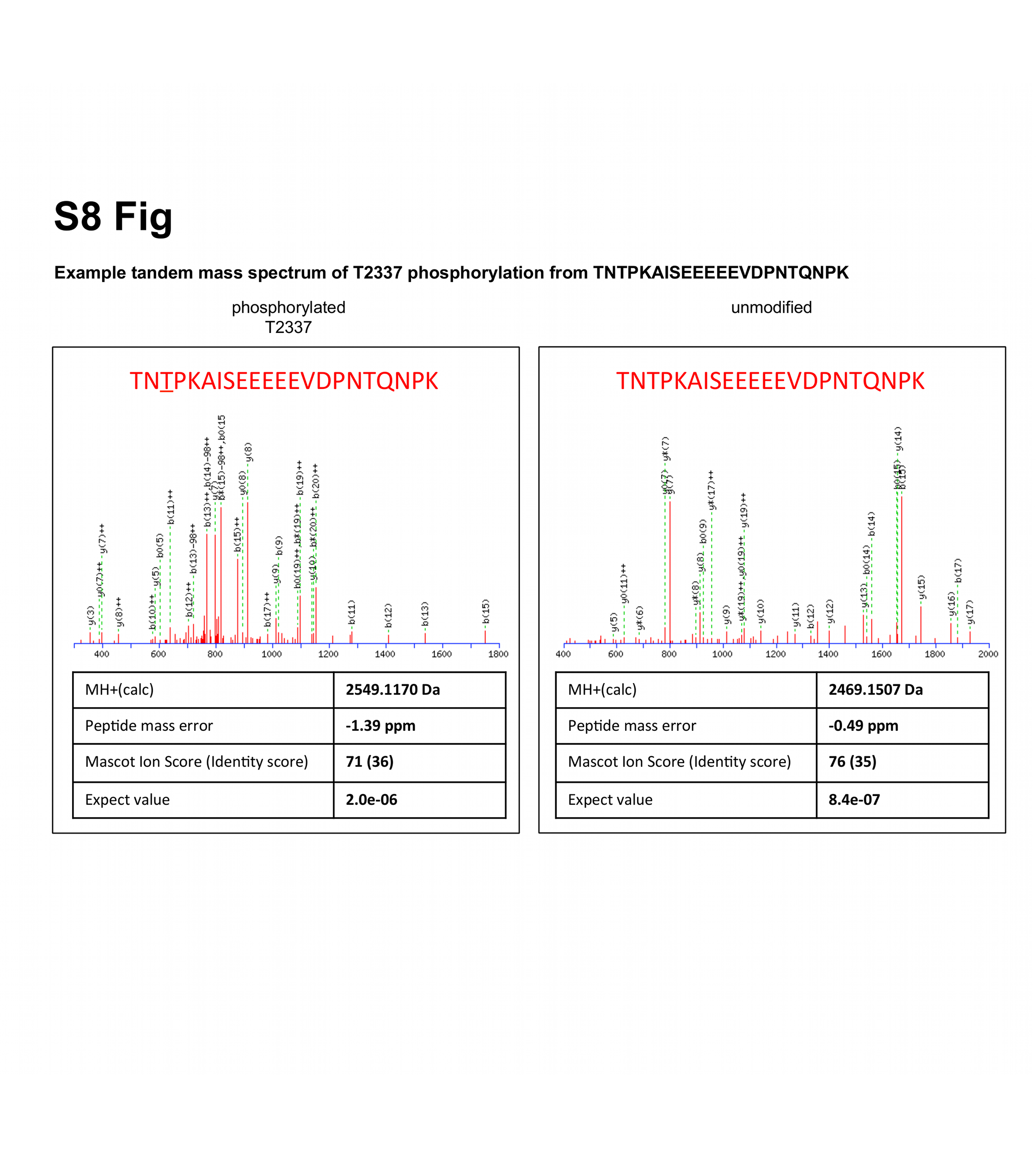

Supplement: S8 Fig — (TIF) [file pone.0121055.s008.tif]

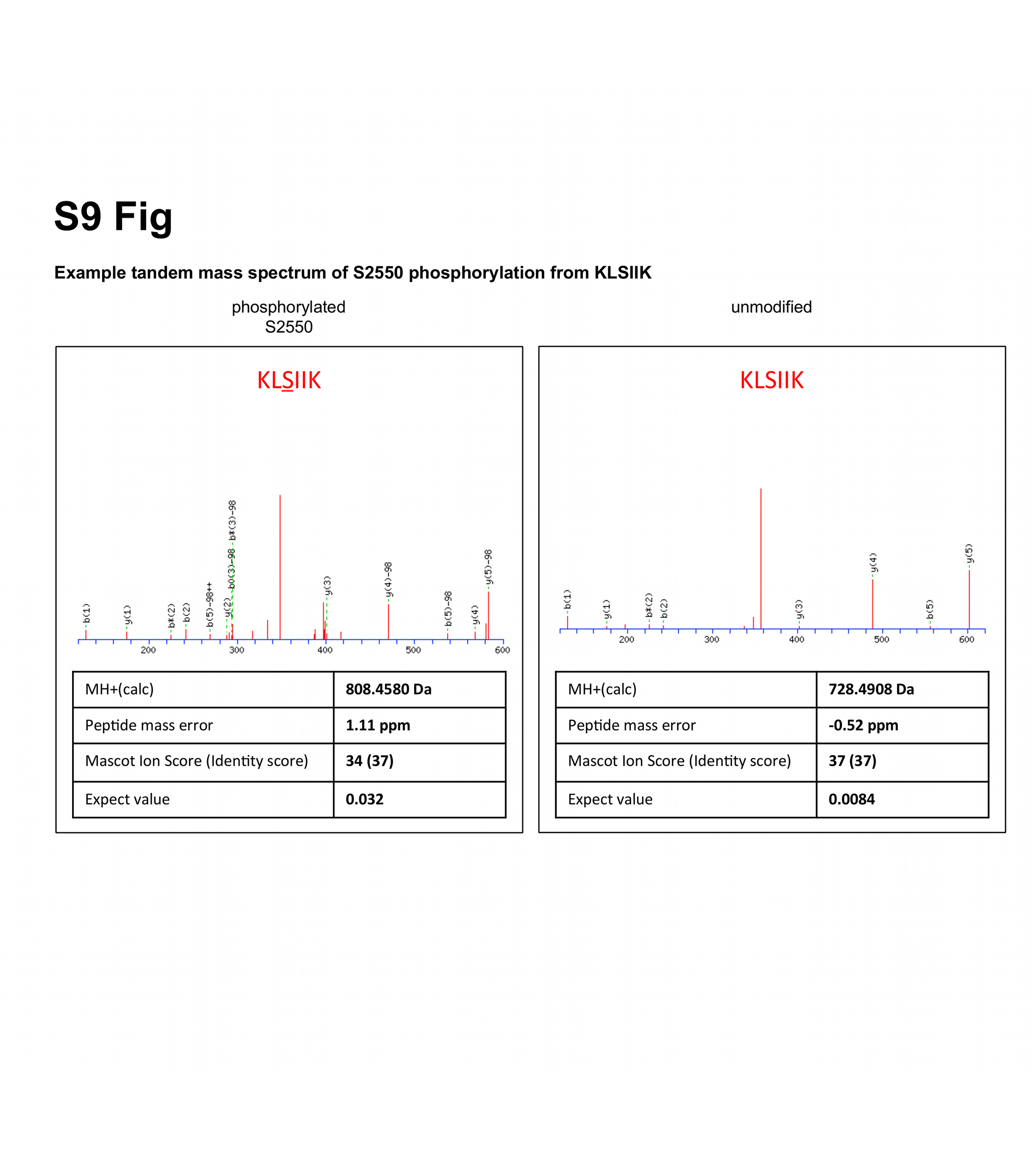

Supplement: S9 Fig — (TIF) [file pone.0121055.s009.tif]
